# Revisiting the Q_A_ model of chlorophyll-a fluorescence induction: new perspectives to monitor the photochemical activity and structural dynamics of photosystem II

**DOI:** 10.1007/s11120-025-01178-x

**Published:** 2025-10-23

**Authors:** Győző Garab

**Affiliations:** 1 Department of Physics, Faculty of Science, University of Ostrava, Ostrava, Czech Republic; 2 Institute of Plant Biology, HUN-REN Biological Research Centre, Szeged, Hungary

**Keywords:** Chlorophyll-a fluorescence, Conformational transitions, Dielectric relaxation, *F*_v_/*F*_m_, Photosystem II, Q_A_ model

## Abstract

The technique of chlorophyll-a fluorescence induction (ChlF) is widely used in plant biology. The ‘mainstream’, so-called Q_A_ model of ChlF, posits that the reaction centers (RCs) of Photosystem-II (PSII) exist in two states, quenched (*F*_o_) or open (PSII_O_), and unquenched (*F*_m_) or closed (PSII_C_), containing the primary quinone acceptor, Q_A_, in oxidized and reduced state, respectively; and that the quantum yield of PSII photochemistry of a dark-adapted sample is *Y(II)* = *F*_v_/*F*_m_, where *F*_v_=*F*_m_-*F*_o_. The widespread application of ChlF, with user-friendly instruments, and the use of the Q_A_ model, have substantially contributed to our understanding of the operation of the photosynthetic machineries under different environmental conditions. However, recent experimental data – multiple light-induced fluorescence increments in PSII_C_; the complex, pH and temperature dependent kinetic and spectral features of key ChlF parameters; twith enhanced stabilization of the charges – cannot be reconciled with the Q_A_ model. These features are explained by subtle conformational transitions driven by stationary and transient electric fields and associated dielectric relaxation processes. This interpretation, while invites further studies, places the hitherto unknown structural and functional plasticity of the RC matrix in the context of its physiological significance.

## Introduction

The non-invasive technique of chlorophyll-a (Chl-a) fluorescence is widely used in plant biology, with broad range of applications in basic research as well as in stress physiology, agriculture and ecology. The induction kinetics of Chl-a fluorescence (hereafter referred to as ChlF) of green leaves was discovered and correlated with the CO_2_ fixation nearly a century ago (Kautsky and Hirsch 1931). In later decades, it was thoroughly documented that ChlF is exhibited by all functionally active oxygenic photosynthetic organisms. It has also been clarified that it is associated primarily with the photochemical activity of Photosystem II (PSII), but the kinetic traces depend on the molecular architecture and functioning of the entire photosynthetic machinery and are influenced by physiological and environmental factors. Accordingly, ChlF contains several kinetic components and occurs over a range of timescales. (For historical aspects of ChlF and different nomenclatures of the kinetic components, the reader is referred to the review of Govindjee (1995); and for the use of Chl-a fluorescence in photosynthesis research, to the book edited by Papageorgiou and Govindjee (2004).)

Interpretation of ChlF – under different experimental and environmental conditions and in isolated thylakoid membranes (TMs) and PSII preparations, as well as in organisms from cyanobacteria and unicellular algae to vascular plants in different habitats and over timescales spanning orders of magnitudes – had been a challenging task. However, thanks to the pioneering works of some of the giants of photosynthesis research, who introduced several simple and elegant assumptions (see below), ChlF transients could be interpreted acceptably at all levels of their complexity. The widely accepted, ‘mainstream’ model – the so-called Q_A_ model, which albeit was never free of controversies – was immensely successful and greatly advanced our understanding of the molecular mechanisms of the photosynthetic light energy utilization. The ingenuity of the Q_A_ model might be sensed by the fact that its basic tenets were set in the 1960s and 1970s – in an era when our knowledge about the molecular architecture and fundamental processes of the photosynthetic machinery was much less advanced than today.

PSII, or water-plastoquinone oxidoreductase, is a large multi-subunit pigment-protein complex embedded in the TMs of oxygenic photosynthetic organisms. Each monomer of the homodimeric core complex (CC) of PSII contains the RC, constituted with the D1 and D2 proteins and the cytochrome *b*_559_, the integral antenna proteins CP43 and CP47, the oxygen-evolving complex (OEC), and several additional protein subunits and cofactors (Shen 2015; Shevela et al. 2023; Hussein et al. 2024). PSII-CC is the smallest unit of the oxygenic photosynthetic apparatus that is capable of performing all basic photophysical and photochemical functions of PSII (Yang et al. 2022). PSII supercomplexes also contain peripheral membrane-embedded light harvesting antenna proteins (Croce and van Amerongen 2011).

PSII uses light energy to oxidize water and, in concert with Photosystem I (PSI), delivers electrons for the conversion of carbon dioxide to sugars. In open-state PSII (PSII_O_), which is capable of generating stable charge separation, the primary charge separation is initiated with the formation of P_680_^**•**+^Pheo^**•**–^, which occurs along the D1 branch (Romero et al. 2017). (Here, P_680_, following the conventional notations, refers to the primary electron donor from which charge separation starts, irrespective of its molecular identity.) The charge separation is stabilized by the re-oxidation of Pheo^**•**–^ by electron transfer to Q_A_, which occurs on a timescale of ~ 300 ps. P_680_^**•**+^ is then re-reduced by electron donation from the nearby redox-active tyrosine, forming the neutral tyrosyl radical TyrZ^**•**^(H^+^), which is then reduced by the Mn_4_CaO_5_ cluster, leading to the S_2_(^+^) state of the OEC; these proton-coupled electron transfer steps proceed on timescales between several nanoseconds and tens of microseconds (Siegbahn 2013; Zaharieva and Dau 2019). The stabilization of the charge-separated state leads to the formation of PSII_C_, the closed state of PSII with Q_A_ reduced. This state persists for several hundreds of µs until the electron is transferred from Q_A_^−^ to Q_B_, and is accompanied by the advancement of the S-states of the OEC. The secondary electron transfer from Q_A_^−^ to Q_B_ re-opens the PSII_C_; this can be blocked, by PSII inhibitor molecules, such as diuron (DCMU, *N´*-(3,4-dichlorophenyl)-*N*,*N*-dimethylurea), and at cryogenic temperatures (< 230 K) (Garbers et al. 1998). PSII_C_ is not capable of forming stable charge separation. Additional excitations of PSII_C_ generate rapidly recombining P_680_^**•**+^Pheo^**•**–^ radical pairs (Sipka et al. 2019).

## Tenets and key features of the Q_A_ model

### Tenet (1): Two states of PSII

The “RCs of PSII exist in two states, quenched and unquenched, containing oxidized and reduced quenchers, Q and QH”. “The excitation energy […] is trapped if the center is in oxidized state” but the „reduced reaction center, QH, does not trap the excitation energy and thus does not quench the fuorescence“ (Duysens and Sweers 1963). In terms of ChlF, the quenched and unquenched state display minimum and maximum fluorescence levels, *F*_o_ and *F*_m_, respectively. Accordingly, “in order to reach *F*_m_, it is necessary, and sufficient, to have Q_A_ completely reduced in all the active PSII centers” (Stirbet and Govindjee 2012).

### Tenet (2): Y(II) = *F*_v_/*F*_m_

To determine the photochemical quantum yield of a dark-adapted PSII, in this paper denoted as Y(II), Butler (1978) derived the widely used equation *Y(II)* = *F*_v_/*F*_m_, by employing a “simple photochemical model in which fluorescence (F), photochemistry (P), and nonradiative decay (D) all compete for the excitation energy in the antenna chlorophyll of PS II by first order rate processes”; and positing that “when the PSII reaction centers are all open, the yield of photochemistry is maximal and the yield of fluorescence is at a minimum. However, when the reaction centers are closed because the primary, electron acceptors are reduced, [Y(II)] goes to zero and the yield of fluorescence increases to a maximum.” Conclusively, because the sum of the quantum yields of all these pathways must be 1.0, by measuring the fluorescence quantum yields with active and inactive photochemistry, the photochemical yield can readily be calculated.

### A co-dependent quantity, Y(NO) = 1-*F*_v_/*F*_m_

Following the same logic as in Tenet (2), an additional quantity, Y(NO), the quantum yield of the so-called constitutive or non-regulatory non-photochemical quenching (npq) of Chl-a fluorescence was introduced to satisfy the condition that the sum of the quantum yields of all competing processes is 1.0. In the presence of regulatory npq: *Y(II) + Y(NPQ) + Y(NO) = 1.0*, and in dark-adapted sample, using Y(II) = *F*_v_/*F*_m_, *Y(NO)* = 1-*F*_v_/*F*_m_ (Genty et al. 1996; Klughammer and Schreiber 2008). Y(NO) is evidently a co-dependent quantity of ChlF, and thus it is not considered as independent tenet of the Q_A_ model.

### Connectivity of PSII RCs as derived from ChlF

The observed sigmoidal (instead of exponential) rise of ChlF, in the presence of DCMU, was assigned to reflect energetic connectivity of PSII RCs, as proposed first by Joliot and Joliot (1964).

## The Q_A_ model scrutinized

As explained in details in a recent review (Garab et al. 2023), the controversial features of the Q_A_ model cumulated in the past decade. (For an earlier stage, focusing on the debated origin of the so-called thermal phase, see Stirbet and Govindjee 2012; Schansker et al. 2014; Kalaji et al. 2014; see also Sect. “Interpretation of ChlF parameters. Applications”). In recent years, it became evident that the parameters of ChlF, using the Q_A_ model, have lost their meanings. Nonetheless, many laboratories continue using the Q_A_ model, which, by today, hinders rather than facilitates our understanding of the physical and molecular mechanisms of the photosynthetic machinery. This is because „feeling like we understand something stops us from asking what it is” (a sentence attributed to Albert Einstein).

In the following paragraphs, I briefly repeat key arguments and literature data which have led to our conclusion that ChlF must be laid on new foundations (Garab et al. 2023); and add recent experimental observations from our laboratory, which cannot be reconciled with Tenets (1) and (2). At the same time, it is pointed out that the proposed physical mechanism of ChlF appears to be suitable to resolve long-standing controversies in the literature. Although presently no exact answer can be given to the question „what it [*F*_v_] is”, in the last sections of this article I will emphasize that ChlF carries physiologically important information on the photochemical activity and structural and functional dynamics of PSII.

### Ad tenet (1): Reduction of Q_A_ explains only a small portion of *F*_v_; additional state, PSII_L_

**Fig. 1:**
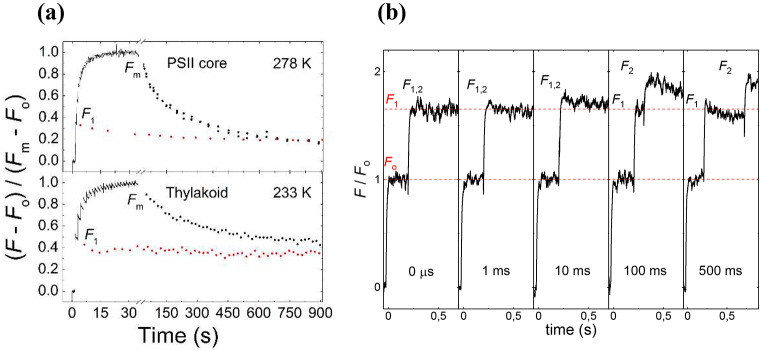
STSF induced Chl-a fluorescence transients of dark-adapted DCMU-treated PSII CC of *Thermostichus (T) vulcanus* and spinach TMs. Panel (**a**) shows the ChlF transients elicited by trains of STSFs and decay kinetics from the *F*_1_ and *F*_m_ levels (reproduced from Magyar et al. 2018). Panel (**b**) illustrates that to generate the *F*_1_-to-*F*_2_ rise on PSII CC requires long Δ*τ* waiting times; these traces were recorded at 193 K (reproduced from Magyar et al. 2023)

In DCMU-treated samples, the first STSF (single-turnover saturating flash), generating PSII_C_, was shown to induce *F*_1_(< *F*_m_) and additional excitations were required to reach *F*_m_ (Joliot and Joliot 1979). It was discovered more recently that, for completing the *F*_1_-to-*F*_2_-to*-F*_3_…*F*_m_ fluorescence increments, sufficiently long Δ*τ* waiting times are required between consecutive STSFs (Magyar et al. 2018; Sipka et al. 2021) (Fig. 1). (It is to be stressed here that Joliot and Joliot (1979) and Sipka et al. (2021), by using electrochromic absorbance transients, ΔA_515_ and C550, verified that the first STSF generated stable charge separation, with Q_A_ reduced in all the active PSII RCs. Also, it has been shown (Sipka et al. 2019) that, in the presence of DCMU, only the first STSF induces stable charge separation.) Variable fluorescence of PSII_C_ has also been observed in untreated sunflower leaves (Laisk and Oja 2020); and it has been shown that the ‘immediate’ rise of ChlF and that measured at 40 µs are merely 1.8x*F*_o_ and 3x*F*_o_, respectively (Oja and Laisk 2020).

These data have clarified that the reduction of Q_A_ per se offers no or at most only partial explanation for the *F*_o_-to-*F*_m_ rise. Recent experiments, using DCMU-treated PSII CC, also revealed that the *F*_1_ level and the half-waiting time (Δ*τ*_1/2_) of ChlF are remarkably sensitive to pH between 5.0 and 8.0, and between 6.5 and 7.0, in particular, suggesting the role of protonatable residues with pK values in this range. (Magyar et al. 2025). In contrast, the photoreduction of Q_A_ between pH 6.0 and 8.0 is essentially pH independent (Demeter et al. 1985; Krieger et al. 1995). Also, whereas the photoreduction of Q_A_ is an activationless process and, in fact, accelerated below 100 K (Kirmaier et al. 1985), the *F*_1_ level decreases dramatically with lowering the temperature (Magyar et al. 2018; Sipka et al. 2021). It has also been revealed that, in addition to PSII_O_ and PSII_C_, PSII can assume light-adapted charge-separated (PSII_L_) state with enhanced stabilization of the charges compared to PSII_C_ (Sipka et al. 2021). Features of PSII_L_ resemble the light-adapted charge-separated state identified earlier in purple bacterial RCs (reviewed by Sipka et al. 2022; for more details, see below).

### Ad tenet (2): Y(II) ≠ *F*_v_/*F*_m_

**Fig. 2:**
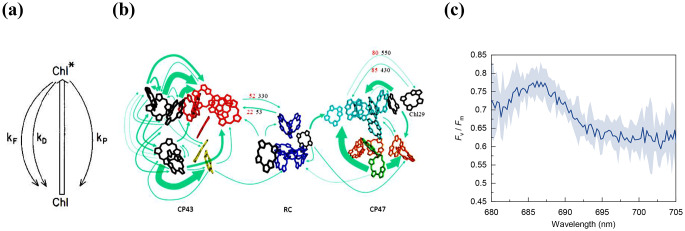
Illustrations to the Section ‘Ad Tenet (2)’. Panel (**a**): Butler (1978) explained the *F*_o_-to-*F*_m_ rise by using Chl-a as a “simple photochemical model” of PSII. Panel (**b**): Shibata et al. (2013) employed a structure-based model to describe the variations of Chl-a fluorescence emission spectra of PSII CC between 5 and 180 K. Panel (**c**): Sipka et al. (2021) have shown that in PSII CC at 80 K F695 > F685 in all emission spectra taken at or near *F*_o_, *F*_1_ and *F*_m_, but the *F*_v_/*F*_m_ spectrum is dominated by the emission from CP43 (F685). Panels (**a**), (**b**) and (**c**) are reproduced from Butler (1978), Shibata et al. (2013) and Sipka et al. (2021), respectively

Tenet (2) of the Q_A_ model (Butler 1978) rests on the implicit assumptions that “none of the rate constants change as the traps go from open to closed, and that all the fluorescence that is observed in both the *F*_o_ and *F*_m_ states come from a homogeneous system in which all chlorophyll excited states are equivalent” (Blankenship 2021). Numerous data show that the scheme of de-excitation pathways occurring in monomeric Chl-a molecule (Fig. 2a) is not applicable on PSII. The analogy omits important structural and functional details – which were not known in 1978. In fact, it has been shown that charge separation, stabilization, and protein relaxation in PSII_C_ follows a complex pattern (Szczepaniak et al. 2009) and that the de-excitation pathways in PSII CC at cryogenic temperatures are governed by a number of rate constants and interplays between the RC and the CP43 and CP47 antenna complexes (Shibata et al. 2013) (Fig. 2b). It has also been shown that the rate constants associated with the multicomponent fluorescence decay of DCMU-treated PSII CC at 5 °C change substantially between PSII_C_ and PSII_L_ (Sipka et al. 2021). Further, the pronounced selective participation of F685 in the *F*_v_ of PSII CC at 80 K (Sipka et al. 2021) (Fig. 2c) has shown that the presupposition that “all chlorophyll excited states are equivalent” is untenable. Most recent experiments using time-resolved ChlF spectroscopy on PSII CC at 10 K showed that sizeable *F*_v_ was displayed only by the F685 band, which also dominated the *F*_v_/*F*_m_ decay-associated emission spectrum (Singhal S, Sipka G, Akhtar P, Sharma A, Han W, Li X, Santabarbara S, Han G, Shen J-R, Garab G, Fülöp JA, Lambrev PH – unpublished). It is to be noted here that in microalgal cells and isolated plant PSII (BBY) membranes at physiological temperatures, the band-shape of the *F*_V_ spectra have been shown to display no siginificant excitation-wavelength dependence (Santabarbara et al. 2019) – which is explained by the rapid thermal equilibration of the excitation energy in PSII supercomplexes at room temperature (Jennings et al. 1993; Dau and Sauer 1996). The pathways of the excitation energy in PSII_C_ and PSII_L_ (super)complexes might be possible to depict by using 2D electronic spectroscopy (Nguyen et al. 2024).

### Y(NO) ≠ 1-*F*_v_/*F*_m_

Because, as shown above, *Y(II)* ≠ *F*_v_/*F*_m_, the co-dependent quantity Y(NO) loses its grounds. To illuminate this point, I recall the results of two easy-to reproduce experiments: (i) we recorded ChlF transients on DCMU-treated PSII CC using trains of either single or simultaneously-fired double STSFs (Garab 2024); (ii) we used intact pea leaves and induced fast ChlF (so-called O-J-I-P) transients by employing intense long flashes with photon flux densities between 3,000 and 15,000 µmol photons m^− 2^ s^− 1^ (Schansker et al. 2011). In these experiments the *F*_v_/*F*_m_ ratios reached their maxima of about 0.8; hence, because of the absence of detectable npq, Y(NO) assumed its minimum value of about 0.2, satisfying the conditon that Y(II) + Y(NO) = 1.0. Evidently, however, the Y(II) and Y(NO) quantum efficiencies should not be independent of the variations of the excitation intensities by a factor of 5.

To avoid controversies of this kind, besides recording ChlF with sub-µs time resolution (Klughammer et al. 2024), independent experiments would be needed to determine the true value of Y(NO) and to shed light on the fate and effects of dissipated excitations; this may need ultrafast vibrational spectroscopy techniques (Ruan et al. 2025). With regard to Y(II), it has been shown, in reasonable agreement with the *F*_v_/*F*_m_ data, that the quantum efficiency of PSII charge separation – as calculated using fluorescence lifetime measurements, via determining “the average lifetime of an excited state in PSII in the presence and (hypothetical) absence of charge separation” – depends on the size of the antenna (Wientjes et al. 2013). In TMs isolated from low-light and high-light grown *Arabidopsis thaliana* Y(II) was found to be 84 and 91%, respectively. This is because the average lifetimes can be considered as the sum of the trapping time of an equilibrated system and the migration time (Wientjes et al. 2013). In the absence of large antenna systems, it is generally agreed that “in photosynthetic reaction centres [the] solar excitation energy is converted into a stable pair of separated charges with close to 100% efficiency” (Romero et al. 2017).

### Sigmoidicity without connectivity

It has been pointed out by Vredenberg (2011) that the sigmoidicity of *F*_v_ can readily be explained by overlapping exponentials. In fact, marked sigmoidicity of *F*_v_ has been observed on isolated DCMU-treated PSII CC dimers and monomers (Sipka et al. 2021) – evidently due to the involvement of multiple consecutive excitations and the stepwise fluorescence increments. It is to be noted that these observations do not rule out the occurrence of connectivity between PSII units.

## Origins of *F*_v_, physical mechanisms, applications, perspectives

As clarified above, the fundamental parameters of ChlF as derived from the Q_A_ model have lost their physical meanings. At the same time, a fresh view on ChlF may open new perspectives to uncover hitherto unknown or sparsely examined characteristics of PSII. In fact, experimental data obtained in the past few years have shown that ChlF carries far more information about the composition, functional activity, conformational states and structural dynamics of PSII than previously thought. To retrieve reliable information on these features and to apply ChlF to physiologically important cases, requires cautious considerations; and to find the correct answers, ChlF may have to be combined with complementary techniques.

In the following sections, first, I will uncover the complex features of *F*_v_, then, discuss the proposed physical and molecular mechanisms and their relevance to physiology. The review will be completed with short concluding remarks.

### Components of *F*_v_

It is now clear that the *F*_o_-to-*F*_m_ rise, i.e. *F*_v_, contains multiple kinetic components and that they are affected by a range of different factors. This conclusion is based on several well-established facts that are listed below.

**Fig. 3:**
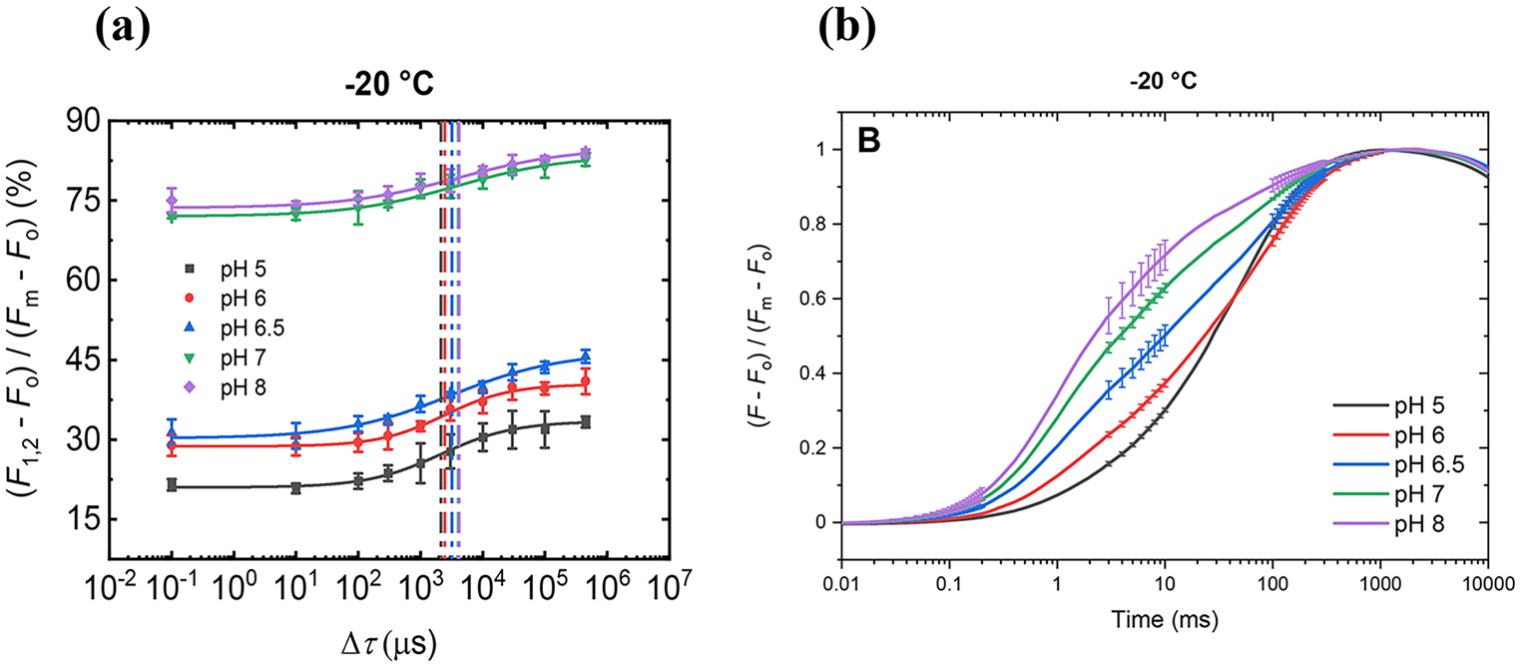
Chl-a fluorescence transients of dark-adapted DCMU-treated PSII CC at different pHs recorded at -20 °C (Magyar et al. 2025). Panel (**a**): double STSF-induced *F*_1_-to-*F*_2_ increment as a function of Δ*τ*; the vertical lines mark the Δ*τ*_1/2_ values. Panel (**b**): double-normalized transients elicited by a long flash – this is to show the pH dependency of *F*_v_, using the more conventional measuring protocol of ChlF. (These figures are reproduced from Magyar et al. 2025)

(i) As shown first by Joliot and Joliot (1979), *F*_m_ cannot be reached upon closing all the active RCs by reducing Q_A_; and to reach *F*_m_, additional STSFs are required, with sizeable Δ*τ*_1/2_ between them (Magyar et al. 2018) (see Fig. 1). These data clearly indicated the participation of at least two distinct processes in *F*_v_. The fact that approximately the same Δ*τ*_1/2_ values were obtained for all *F*_j_-to-*F*_j+1_ steps tested (j = 1,2,3,4) suggests that the nature of the physical mechanism in later steps remains the same (Magyar et al. 2023). Note that to complete the so-called photochemical (O-J) phase by intense long flashes also uses multiple excitations of each RC (Neubauer and Schreiber 1987; Lazar and Pospisil 1999).
(ii) The presently available data suggest that the magnitude of fluorescence rise that is associated directly with the reduction of Q_A_ may not be larger than about 20% of the total rise. This can be inferred from STSF-induced *F*_1_ values in PSII CC at low pH (< 7.0) and cryogenic temperatures (Magyar et al. 2018, 2025; Sipka et al. 2021) (Fig. 3a). Also, the relative amplitude of the first exponential component of the long-flash induced ChlF in PSII CC at pH 6.0 at -20 °C (cf. Figure 3b) and − 60 °C (Magyar et al. 2025) was < 20%. These data are consistent with earlier experiments, using DCMU-treated pea leaves, which have revealed that the fastest kinetic component of ChlF, contributing with 19.6% to the the O-to-P rise, is nearly temperature independent between 30 and − 100 °C, strongly suggesting its origin from the photochemical phase (Schansker et al. 2011). Note that the facts that the *F*_1_ rise varies strongly with the temperature and the pH suggest that *F*_1_ has sub-ms components, with no direct correlation with the reduction of Q_A_. Indeed, Oja and Laisk (2020) has shown that in leaves of vascular plants, the ‘immediate’ rise (1.8x*F*_o_) was followed by a rise to about 3x*F*_o_ in 40 µs. Similar increase of the Xe-flash induced fluorescence level from 60% of *F*_m_ (3.1x*F*_o_) at 30 µs to about 90% of *F*_m_ (4.2x*F*_o_) at 1 ms had earlier been reported using DCMU-treated intact spinach chloroplast measured at room temperature (Schreiber and Krieger 1996).

(iii) It was shown that at cryogenic temperatures (< 230 K) the *F*_1_ rise of TMs and PSII CC remained nearly stable in the dark for > 15 min; in contrast, *F*_m_ displayed marked temperature-dependent relaxation kinetics in the dark, allowing the determination of activation energy of these transitions. These data provided evidence for the involvement of conformational changes associated with the *F*_1_-to-*F*_m_ rise elicited by a train of STSFs (Magyar et al. 2018).
(iv) To elucidate the nature of the photochemical processes associated with the multistep *F*_1_-to-*F*_m_ rise, 819 nm absorbance transients were performed on DCMU-treated PSII CC. The multiphasic decay kinetics after the first STSF was shown to originate from the re-reduction of P_680_^**•**+^ (Sipka et al. 2019). In particular, the fastest decay component (∼20 ns) was attributed to proton-coupled electron transport from TyrZ, and the slower components indicated relaxation processes involving hydrogen bond networks that shift the equilibrium P_680_^**•**+^TyrZ↔P_680_TyrZ^**•**^(H^+^) to the right and reduction of TyrZ^•^(H^+^) by the OEC (Schlodder et al. 2015). In contrast, upon the second and consecutive, or repetitive STSFs, the transients decayed with lifetimes of about 2 ns (∼60%) and 10 ns (∼30%), due to the rapid recombination of the primary radical pairs (Sipka et al. 2019).
(v) The Δ*τ*_1/2_ half-waiting time of the *F*_1_-to-*F*_2_ transition has been shown to depend on the lipid content of the RC matrix (Magyar et al. 2022, 2024) – testifying that Δ*τ*_1/2_ is a physiologically important parameter that characterizes the structural plasticity / dynamics of PSII CC.
(vi) Recent experiments on DCMU-treated PSII CC revealed strong pH dependence of Δ*τ*_1/2_ and of the *F*_o_-to-*F*_1_ rise, clearly demonstrating the involvement of proton-coupled electron transfer processes, and thus pointing to the prominent role of the donor side of PSII in ChlF (Magyar et al. 2025). On the whole, this finding is in harmony with earlier literature data on the oscillation-4 of the Chl-a fluorescence, with marked maxima in the S_2_ state (Delosme and Joliot 2002; Ananyev and Dismukes 2005; see also Klughammer et al. 2024).
(vii) The *F*_1_ and *F*_m_ fluorescence emissions of PSII CC at 80 K have been shown to display distinct spectral distributions, with the corresponding *F*_v_/*F*_m_ spectrum dominated by F685 (Sipka et al. 2021) (see Fig. 2c). As mentioned above, similar, more profoundly asymmetric participation of CP43 (F685) in *F*_v_, with no or only very little contribution from CP47 (F695), was shown by decay-associated emission spectra at 10 K (Singhal S, Sipka G, Akhtar P, Sharma A, Han W, Li X, Santabarbara S, Han G, Shen J-R, Garab G, Fülöp JA, Lambrev PH).

### Multiple stationary fluorescence levels between *F*_o_ and *F*_m_

ChlF experiments using trains of STSFs allowed us to conclude that PSII units display a range of discrete *F*_i_ fluorescence levels between *F*_o_ and *F*_m_ (Magyar et al. 2018, 2025; Sipka et al. 2021) (i = 1,2,3,…) (see also Fig. 1a). Further, the number of discrete steps (*N*) induced by a train of STSFs has been shown to increase dramatically by decreasing the temperature, and can be very large, several dozens and a few hundreds (Sipka et al. 2021). It is important to stress that each *F*_i_ level represents a (quasi-)stationary state, which, when using a ~ 1 ms time-resolution, neither increases nor decreases upon using two, synchronously fired STSF (Magyar et al. 2018; Sipka et al. 2021). (It is important to stress that these single- vs. double-STSF experiments rule out sizeable heterogeneity of the RCs.)

A plausible explanation on the observations outlined above that the multiple discrete levels of *F*_i_ originate from slightly different physico-chemical environments of the RC matrix; and that the consecutive *F*_i_-to-*F*_i+1_ increments reflect conformational transitions generated by consecutive STSFs. These transitions might fine-tune the rate constants that govern the fluorescence emission of PSII CC (Shibata et al. 2013). The high number of *F*_i_ states strongly suggests that the rate constants may assume virtually any value between their extremes; in terms of fluorescence, between *F*_o_ and *F*_m_ or between *F*_1_ and *F*_m_. Note that this interpretation is based on the modulation of fluorescence yield by physico-chemical factors in the RC matrix and in PSII CC, rather than attributed to different degrees of light-induced unquenching of Chl-a fluorescence. (For possible effects of quenchers under our experimental conditions and time resolution (~ 1 ms), see Magyar et al. 2018); these authors emphasize that, if quenchers are involved, consecutive STSFs do not generate but rather eliminate them).

Here, I would like to add a brief comment on the convention of interpreting ChlF in terms of quenching (and unquenching or release of quenching). The conventional terminology of photochemical quenching, originally, referred to the (assumed or observed) action of quencher molecules on Chl-a fluorescence and on primary photochemistry: the hypothetical Q of Duysens and Sweers (1963), which was identified later as Q_A_, and dibromothymoquinone of Kitajima and Butler (1975) – see Tenets (1) and (2). Using this convention, we should phrase the multiplicity of *F*_i_ states as fine-tuning the magnitude of quenching (unquenching) of PSII fluorescence as compared to *F*_m_ (*F*_o_). Note that, strictly speaking, *F*_m_ is quenched compared to Chl-a in solution. Because of the multiplicity of *F*_i_, I propose to use the term modulation of fluorescence yield, rather than the terms quenching / unquenching.

The terminology of quenching suits better the photoprotective mechanisms of NPQ (for recent review, see Zuo 2025). In general, in green plants quenchers are generated in the main light-harvesting antenna complexes of PSII (Ruban and Saccon 2022). I wish to note here that, in the absence of (an) identifiable quencher molecule(s), NPQ might also be treated as modulation of Chl-a fluorescence by physico-chemical factors, such as binding of protons to specific residues. It has been hypothesized that such residues may temporarily store ‘excess’ protons, allowing their delayed utilization under conditions favorable for ATP synthesis (Garab et al. 2025). Note also that NPQ is conventionally measured by comparing *F*_m_ levels, with the assumption (Tenet (1) of the Q_A_ model) that “saturation pulse […] causes all the electron acceptors (Q_A_) in PSII to become fully reduced, leading to maximum fluorescence (Fm)” (Zuo 2025). It remains to be tested by independent experiments if intermediary states between PSII_C_ (*F*_1_) and PSII_L_ (*F*_m_) occur during the onset and relaxation phases of NPQ.

The multiplicity of *F*_i_ levels can also be interpreted in a generalized scheme considering radiative versus nonradiative processes. As pointed out by Ostroumov et al. (2014), the quantum yield of Chl-a fluorescence emission of PSII is determined by the rate constants of photochemistry (*PQ* – photochemical quenching, i.e. trapping of the excitation by the RC), fluorescence (*Fl*), intersystem crossing (*ISC*) and internal conversion (*IC*). In PSII_O_, the *k*_PQ_ rate is high and thus the quantum yield of *PQ* is also high (89%); conversely, the yields of *Fl*, *ISC* and *IC* are small, explaining *F*_o_. In PSII_C_ the quantum yield of *PQ* decreases to 76% and the Chl-a fluorescence rises to *F*_m_ (Ostroumov et al. 2014. Note that, in contrast to the original assumptions used in Tenets (1) and (2), the quantum yield of *PQ* in PSII_C_ does not drop to zero because the primary charge separation, the formation of the P_680_^**•**+^Pheo^**•**–^, still occurs (at least as long as the P_680_^**•**+^ is re-reduced), as also demonstrated by Sipka et al. (2019). To explain the additional state, PSII_L_ and the transitional states producing *F*_i_ levels within the frameworks of the theoretical model presented by Ostroumov et al. (2014), we have to assume that the rate constants (*k*_PQ_, *k*_Fl_, *k*_ISC_, *k*_IC_) are adjusted upon the PSII_C_-to-PSII_L_ transition; which can be envisioned, e.g., via modulating the interactions between the RC and the core antenna complexes (Shibata et al. 2013). (Note also that Ostroumov et al. (2014) introduces a state, which might be called as PSII_NPQ_, in which the quantum efficiency of NPQ dominates over that of *PQ*.) In general, the excitation energy transfer rates and different fluorescence parameters depend on the local refractive index / dielectric constant; which may vary dramatically upon nearby protonation / deprotonation events (Lakowicz 2006). In principle, events of this kind and the associated (evidently subtle) structural changes can be identified by advanced techniques of time-resolved spectroscopy and structural biology.

### Physical mechanism(s)

Concerning the physical mechanism(s) associated with ChlF – beyond the effects attributable directly to the reduction of Q_A_ – relevant experimental data have been gathered in the past years. Before giving an account on them, it is interesting to recall data on light-induced conformational changes in purple bacterial reaction centers, bRCs, the ancestors of PSII RCs.

Upon illumination of closed bRCs with continuous light, long-lived charge-separated states have been identified, in which the rate of charge recombination between the photooxidized primary donor (P^+^) and the photoreduced quinone acceptor (Q_A_^−^) is slowed down by up to three orders of magnitude (Abgaryan et al. 1998; Andréasson and Andréasson 2003; Goushcha et al. 2003; Lukashev et al. 2014; Sipka et al. 2018; Deshmukh et al. 2011b; Malferrari et al. 2014). Remarkably, further stabilization of the charge separated states could be achieved in the presence of added lipids, in proteoliposomes (Deshmukh et al. 2011a). A theoretical model has shown that the gradual formation of the light-adapted conformational state can only be observed after repeated excitation of the sample (Barabash et al. 2002). The formation of the light-adapted charge-separated state in bRCs is thought to originate from conformational transitions associated with dielectric relaxation processes (Deshmukh et al. 2011b; Malferrari et al. 2013). Recently, Allen et al. (2022) have identified hydrogen-bond networks and bound water molecules, which have been proposed to be responsible for the very slow charge recombination kinetics observed after continuous illumination of bRCs.

**Fig. 4:**
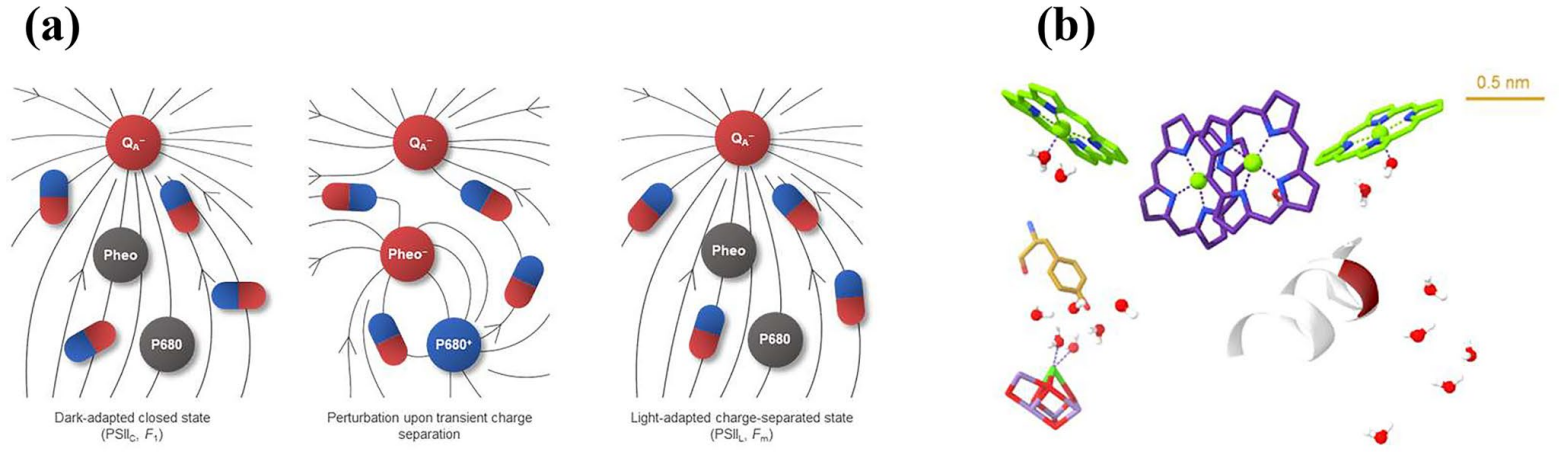
A proposed physical mechanism and molecules potentially involved in the gradual formation of PSII_L_ from PSII_C_. Panel (**a**): Schematic illustration of the dielectric relaxation processes in the presence of stationary and transient electric fields (reproduced from Sipka et al. 2021). Panel (**b**): Cofactor structures and bound water in the RC *T. vulcanus* (pdb:5GTH) (reproduced from Sipka et al. 2022)

The above data on bRCs offer explanation on key observations in DCMU-treated PSII CCs, where a 3-fold increase was observed in the recombination half-time between the S_2_ state of the OEC and Q_A_^−^ (Sipka et al. 2021). Similar to the charge stabilization in bRCs, dielectric relaxation of the protein matrix of PSII may explain the stabilization of charges in PSII_L_ compared to PSII_C_ (Fig. 4a). The existence and features of the novel ChlF parameter, Δ*τ*_1/2_, characteristic of the structural dynamics of the RC matrix, was also explained in terms of dielectric relaxation processes. (It is an interesting question whether or not bRC also requires Δ*τ*_1/2_ in the formation of their light-adapted state).

The conformational changes in PSII are evidently hindered by the rigidity of the protein matrix of the RCs; the structural rigidity becomes more pronounced at cryogenic temperatures (Magyar et al. 2023). With respect to the polarizable groups, that are prone to participate in dielectric relaxation even at low temperatures, we inspected the scheme of Allen et al. (2022) on bRCs, and found a very similar hydrogen bond network and bound water molecules in PSII (Sipka et al. 2022) (Fig. 4b). In broad terms, the presence of these polarizable groups on the donor side of PSII RC explains the protonation dependencies of *F*_1_ and Δ*τ*_1/2_ (Magyar et al. 2025). The conformational transitions were proposed to be driven by stationary and transient electric fields due to the stable charge separation (S_2_^(+)^Q_A_^−^) and the rapidly recombining radical pair, P_680_^**•**+^Pheo^**•**–^, respectively (Sipka et al. 2021). The emergence and disappearance of the transient field is proposed to represent a perturbation in the dielectric matrix, which, after several (or a large number of) repetitions gradually reaches the optimal conformational / dielectrically relaxed state suited to the stationary field (cf. Figure 4a). In addition, fast local thermal transients, similar to those detected in light-harvesting antenna complexes (Cseh et al. 2000; Gulbinas et al. 2006), were hypothesized to facilitate the conformational changes by transiently ‘melting’ the rigid structure of the RC matrix. Further, lipids were proposed to act as ‘mechanical transducer’ molecules, which warrant the plasticity / structural dynamics of the RC matrix (Magyar et al. 2024).

### Interpretation of ChlF parameters. Applications

As it has been pointed out earlier (Garab et al. 2023), one of the main controversial features of the Q_A_ model, since the 1960s, had been that upon using an intense, long (~ 1 s) rectangular flash excitation on dark-adapted untreated TMs, algal cells or leaves, the fluorescence maximum (P) cannot be reached in the photochemical (O-J) phase; P is reached only in the thermal phase, which is characterized by the J–I–P rise (Stirbet and Govindjee 2012). The controversy is most striking by taking into account that in the photochemical phase, which (under the usually employed photon flux density of 3000 µmol m^–2^ s^–1^) is completed in about 2 ms, each RC receives several excitations (cf. Neubauer and Schreiber 1987; Lazar and Pospisil 1999). Note that the same controversy exists in the presence of DCMU: excess quanta are required to generate the O-to-P rise, too (see also the STSF experiments discussed above).

The fundamental controversy of ChlF within the frameworks of the Q_A_ model, outlined above, can be resolved, at least qualitatively, by taking into account that the *F*_o_-to-*F*_m_ (O-to-P) fluorescence rise requires not only multiple excitations but also relatively long Δ*τ* waiting times between excitations. Since, in the physiological temperature range, Δ*τ*_1/2_ is commensurate with the halftime of the Q_A_-to-Q_B_ electron transfer, which reopens the RC, the two processes compete with each other (Magyar et al. 2018; Sipka et al. 2021). Further, it must also be taken into account that the electron transfer on the acceptor side of PSII is accompanied with the advancement of the S-states, which is an additional factor modulating the fluorescence yield (Delosme and Joliot 2002; Ananyev and Dismukes 2005). The oscillation-4 of the fluorescence yield, with maxima in S_2_ state and minima in S_3_ and S_4_ states, can be considered as a form of donor-side quenching (Schreiber and Neubauer 1987, Klughammer et al. 2024). The lower yield of the fluorescence with mixed S states, compared to S_2_^(+)^Q_A_^−^, in combination with P_680_^+^, accummulated upon using high intensity excitation (Schansker et al. 2011), may indeed explain the frequently observed dip after the J level (Stirbet and Govindjee 2012).

A detailed mathematical model describing the complex O-J-I-P rise should thus take into account the rate constants of the Q_A_-to-Q_B_ electron transfer and the relevant Δ*τ*_1/2_ values, as well as the number of excitations (N) used and the modulation of the fluorescence yield due to S-state distributions; and probably additional factors, such as the accummulation of P_680_^+^, the redox state of the plastoquinone pool and the photochemical activity of PSI. Note that these factors, with the exception of the Δ*τ* and N, have been shown to affect the complex O-J-I-P transients (Stirbet and Govindjee 2012; Strasser et al. 2004; Kalaji et al. 2014; Klughammer et al. 2024). From a practical point of view, experiments can be designed to vary the Q_A_-to-Q_B_ electron transfer rates, e.g. by varying the temperature (Renger et al. 1993) or using mutants (Lambreva et al. 2025) – and to monitor variations in the magnitude of the O-J phase and/or in the O-J-I-P transients (Schansker et al. 2011; Stirbet and Govindjee 2012; Lambreva et al. 2025). Parallel experiments should be conducted to determine the Δ*τ*_1/2_ values, preferentially in the presence of DCMU (Magyar et al. 2023), but otherwise under the same experimental conditions; further, the distribution of the S-states should also be determined by thermoluminescence (Ducruet and Vass 2009) and/or by O_2_ electrode (Delosme and Joliot 2002). Systematic experiments at subfreezing temperatures, where the secondary electron transfer steps are inhibited (Renger et al. 1993) and the thermal phase disappears (Morin 1964; Stirbet and Govindjee 2012), are also of substantial interest.

Close correlation between the *F*_v_/*F*_m_ ratio and the functional activity of PSII have been thoroughly documented (Genty et al. 1989; Papageorgiou and Govindjee 2004; Strasser et al. 2004; Murchie and Lawson 2013). This correlation appears to be quite robust, indicating that, in general, the high *F*_v_/*F*_m_ ratio can be considered as the hallmark of photochemically active, healthy PSII. This, however, does not justify to equate the *F*_v_/*F*_m_ ratio with the photochemical quantum efficiency of the RCs because, beside the fact that it would give a false numerical value, it would ignore the structural-dynamic components of *F*_v_ and the gradual light-induced formation of PSII_L_. Also, there are clear examples in which samples with dramatically decreased *F*_v_/*F*_m_ ratios display high rates of oxygen evolution (Vavilin et al. 1999; Treves et al. 2016). These cases may warn researchers to give less credit to the *F*_v_/*F*_m_ ratio, as a sole parameter.

There are numerous publications, in which marked variations in the *F*_v_/*F*_m_ ratio, for simplicity and probably by convention, are interpreted as changes in the photochemical quantum efficiency of PSII. In simple cases, e.g. under photoinhibitory conditions or under heat stress or in the presence of toxic metals, the decline in the *F*_v_/*F*_m_ ratio is readily explained by a heterogenous population of the RCs, a mixture of fully active and fully inactive RCs, as in fact proposed by some authors (e.g. Kono et al. 2022; Vass and Cser 2009; Mattila et al. 2023). By providing a value on Y(II) would imply, most certainly against the intention of the authors, that the quantum efficiency of each RC is modulated.

The value of the *F*_v_/*F*_m_ ratio might be lowered due to ‘dead’ fluorescence. As pointed out by Santabarbara et al. (2019) “the excitation/emission dependencies of the *F*_v_/*F*_m_ ratio arise from overlapped contributions from the three independent emissions of PSI, PSII and a fraction of energetically uncoupled external antenna”. Detached or energetically loosely connected antennae, e.g. phycobilisomes, may elevate *F*_o_ but do not participate in the induction – which, as clarified by Remelli and Santabarbara (2018), explains that in whole cells of *Synechocystis* sp. PCC 6803 the *F*_v_/*F*_m_ ratio displays a significant dependency on both the excitation and the emission (detection) wavelength. Diminished supply of the excitation energy from such antenna complexes to PSII RCs might signify the lowering of the photochemical quantum efficiency and/or increased levels of dissipation. Hence, a diminished *F*_v_/*F*_m_ ratio may have an important diagnostic value on the utilizattion of the absorbed light energy.

More interesting cases are in which – e.g. during greening or upon the effects of environmental factors or in mutants and in some organisms in special habitats – the photochemistry and/or the structural dynamics of PSII might exhibit significant variations. These might be manifested in unusual Chl-a fluorescence parameters. Recording ChlF, induced by trains of STSFs, and determining the novel *F*_1_ and Δ*τ*_1/2_ parameters, may shed light on the nature of the structural and functional plasticity of the RC matrices.

With respect to the use of ChlF in intact systems and physiologically important processes and the use of isolated PSII CC, I would like to add the following comments. On one hand, it must be emphasized that albeit quantitative differences might occur between intact and isolated systems, the basic features of ChlF are essentially identical. This can be concluded from different independent sets of experiments: (i) the *F*_v_/*F*_m_ ratios in dark-adapted samples of PSII CC, TMs and leaves of vascular plants are very similar to each other (cf. e.g. Joliot and Joliot 1979; Magyar et al. 2018; Laisk and Oja 2020); (ii) works from the same laboratories have also demostrated, using PSII CC, isolated plant TMs, cyanobacterial cells, and leaves of vascular plants that samples with closed PSII RCs exhibit marked variable fluorescence; (iii) in cyanobacterial PSII CC and plant TMs the dependences of the *F*_1_/*F*_m_ ratios as a function of temperature between 5 °C and − 100 °C followed very similar trends, displaying marked decreases toward cryogenic temperatures (Magyar et al. 2018); (iv) It has been shown that the half-waiting time parameter, Δ*τ*_1/2_, displays very similar temperature dependences in isolated cyanobacterial PSII CC and plant and cyanobacterial TMs (Magyar et al. 2022, 2023) – the observed quantitive differences have been accounted for by differences in the lipid content of their RC matrices (Magyar et al. 2024; see also Sect. “Components of *F*_v_”). On the other hand, as pointed out by Kalaji et al. (2014), in live algal suspensions and intact leaves, heterogeneity of PSII responses might arise due to the optical properties of leaf tissues or of the suspension. ChlF measurements might be affected by intense light scattering, uneven, wavelength-dependent penetration of the exciting beam, strong reabsorption of the fluorescence emission – all of which might be corrected and/or taken into consideration. Additional factors that might influence ChlF data in vivo include chlororespiration, cyclic electron transport, state transitions, diurnal cycles, variations in the CO_2_ fixation rates. These are physiologically important processes, which might be identified and/or monitored by ChlF. Thus, because of their high level of structural and functional complexities, intact, in vivo systems are not suited to clarify the fundamental physical and molecular mechanisms of ChlF. In contrast, isolated subchloroplast particles, are far less interesting from the point of view of physiology, but they allow us to identify basic features of ChlF. In particular, PSII CCs are capable of performing all basic photophysical and photochemical functions of PSII (Yang et al. 2022); their near-atomic resolution structures (Shen 2015; Hussein et al. 2024) and the wealth of stationary and time-resolved spectroscopic data (Shevela et al. 2023) offer an ideal platform to elucidate the fundamental molecular and physical mechanisms underlying the ChlF transients. In turn, uncovering the physical mechanisms may open new perspectives towards the better understanding of the operation and structural and functional plasticity of PSII and its participation in different regulatory mechanisms in different oxygenic photosynthetic organisms. In this, technique of Chl-a fluorescence - with its non-invasive nature, high sensitivity and kinetic capabilities - will most certainly remain an indispensable tool.

## Concluding remarks

The aim of this review article is to call the attention of researchers that – after more than half a century use of the Q_A_ model and many great successes of ChlF – the interpretation of Chl-a fluorescence must be laid on new grounds. This is an unavoidable inference from the facts that the fundamental tenets of the Q_A_ model do not hold true and that the conventionally derived parameters have lost their physical meanings. At the same time, searching for the fine-tuning mechanisms beyond ChlF may open new perspectives towards revealing hitherto unknown features of the structural and functional plasticity of PSII units and to determine their optimal operational conditions. By this means, ChlF, combined with complementary techniques, will most certainly contribute to the better understanding of the mechanisms and regulation of the photosynthetic machineries in different organisms and under different environmental conditions.

## Data Availability

No datasets were generated or analysed during the current study.

## Abbreviations

bRC: Purple bacterial reaction center
CC: Core complex
Chl-a: Chlorophyll-a
ChlF: Chlorophyll-a fluorescence induction kinetics
DCMU: Nꞌ-(3,4-dichlorophenyl)-N, N-dimethylurea
F_o_ minimum: Chl a fluorescence yield in the dark-adapted sample
F_v_ variable: Chl a fluorescence in dark-adapted sample
F_m_ maximum: Chl a fluorescence in dark-adapted sample
npq: Non-photochemical quenching of the first singlet excited state of Chl-a
NPQ: ‘Regulatory’ npq, detectable by monitoring Chl-a fluorescence yield
OEC: Oxygen-evolving complex
P_680_: Primary electron donor of photosystem II
Pheo: Pheophytin
PS: Photosystem
PSII_O/C/L_: Open/closed/light-adapted state of PSII
RC: Reaction center
TM: Thylakoid membrane
STSF: Single-turnover saturating flash
Y(II): Photochemical quantum efficiency of Photosystem II
Y(NO): Quantum efficiency of the so-called nonregulatory or constitutive npq
